# Efficacy and Safety of *Brucea javanica* Oil Emulsion Injection for Treating Gastric Cancer: A Protocol for a Systematic Review and Meta Analysis

**DOI:** 10.1155/2021/5236454

**Published:** 2021-10-15

**Authors:** Luchang Cao, Xinmiao Wang, Heping Wang, Jingyuan Wu, Taicheng Lu, Shixin Li, Jie Li

**Affiliations:** ^1^Department of Oncology, Guang'anmen Hospital, China Academy of Chinese Medical Sciences, Beijing 100053, China; ^2^Graduate College, Beijing University of Traditional Chinese Medicine, Beijing 100029, China

## Abstract

*Introduction*. *Brucea javanica* oil emulsion injection (BJOEI) is an antitumor drug extracted from the traditional Chinese medicinal plant *Brucea javanica*, which has broad prospects as an adjuvant treatment for gastric cancer (GC); however, its efficacy and safety are still controversial. We plan to conduct a systematic review and meta-analysis to summarise the clinical efficacy and safety of BJOEI in the treatment of GC and provide credible evidence for the clinical application and subsequent studies of BJOEI. *Methods and Analysis*. This systematic review will include articles identified by electronically searching the following databases: PubMed, EMBASE, CENTRAL, Web of Science, the Chinese Biomedical Literature Database (CBM), the China National Knowledge Infrastructure (CNKI), Wanfang Database, and Chinese Scientific Journals Database (VIP Database) from inception to 31 July 2021. The primary outcomes of this research will be the clinical total effective rate, performance status, and adverse drug reactions (ADRs). The systematic review will be performed using RevMan 5 software. Finally, we will use the Grading of Recommendations Assessment, Development and Evaluation System (GRADE) to assess the quality of evidence. *Ethics and Dissemination*. Ethical approval is not required for literature-based studies. The results of this systematic review will be published in a peer-reviewed journal. PROSPERO registration number: CRD42021265646.

## 1. Introduction

With the fifth and fourth highest incidence and mortality rates of cancer worldwide, gastric cancer (GC) has become a major threat to human life. According to the 2020 Global Cancer Statistics [1], there were more than 1 million new GC cases and more than 700,000 deaths in East Asia and Eastern European countries. Moreover, GC deaths in China exceeded half of the world's total [2]. Advanced age [3], smoking [4], alcohol consumption [5], intake of smoked and pickled food [6], *Helicobacter pylori* infection [7], gastroesophageal reflux disease [8], malignant anaemia [9], history of gastric surgery [10], and familial inheritance [11] are major risk factors for GC. The development of endoscopy, molecular biology, biomarkers, proteomics, and other disciplines has provided more opportunities for early detection and diagnosis of GC [12–14]. Surgery, chemotherapy, radiotherapy, and molecular targeting are effective methods in the treatment of GC. In the past 100 years [15], the incidence and mortality of GC have been steadily decreasing in most countries worldwide. However, patients diagnosed at an advanced stage may lose the opportunity for surgical treatment. Nonetheless, some patients undergoing surgical treatment still have a 40–70% chance of metastasis after surgery [16, 17]. Moreover, adverse drug reactions (ADRs) in radiotherapy and chemotherapy are common [18, 19]. The overall prognosis of GC remains poor [20]. Therefore, it is important to seek safe and effective supplementary therapies and alternatives treatments for GC.

Traditional Chinese medicine (TCM) has unique advantages in synergism, attenuation of cancer treatment, and comprehensive palliative treatment [21]. Several active compounds in TCM, such as emodin [22], furanocoumarin [23], coumarin [24], *Tabebuia pallida (Lindl.) Miers* [25], and palmitoylethanolamide [26], have been verified to exert anticancer effects through various mechanisms. Studies have shown that various Chinese herbs and active ingredients, such as *Ginkgo biloba* extract, cantharidin, and matrine, have inhibitory effects on the proliferation and invasion of GC [27]. *Brucea javanica* oil emulsion injection (BJOEI) is a new generation of antitumour drugs extracted from the traditional Chinese medicine *Brucea javanica*, mainly including quassinoids and fatty acids. Its main active components are oleic and linoleic acids [28]. Studies have found that BJOE exerts its antitumour effects primarily by inhibiting cell proliferation [29], inducing apoptosis [30], and interfering with cell cycle and energy metabolism [31] and has been widely used in the treatment of a variety of tumour-related diseases [32, 33]. Moreover, it has been shown that BJOE combined with chemoradiotherapy can increase radiotherapy sensitisation [34, 35] and reduce ADRs [36, 37], which can improve the therapeutic effect. However, the 2015 edition of the Chinese Pharmacopoeia reported that *Brucea javanica* has some toxicity. BJOEI can result in allergic reactions, phlebitis, liver damage, kidney damage, and arrhythmia [38, 39]. Therefore, the efficacy and safety of BJOEI in clinical applications are of utmost importance.

Although several systematic reviews have been conducted to evaluate the clinical efficacy of BJOEI on GC, none have assessed the quality of the synthesised evidence and arrived at definitive conclusions. Moreover, several RCTs of BJOEI on GC have been published recently. We plan to conduct a systematic review to objectively evaluate the efficacy and safety of BJOEI in the treatment of GC and assess the quality of the synthesised evidence, considering the latest findings.

## 2. Methods and Analysis

The study protocol was registered in PROSPERO. The registration number for this protocol is CRD42021265646. The protocol was reported according to the Preferred Reporting Items for Systematic Reviews and Meta-Analysis Protocols (PRISMA-P) guidelines. In addition, we will conduct a review following PRISMA statement guidelines (supplementary file S1). Any changes in the full review process were recorded as appropriate. We followed the methods of Shan Gao et al. [40].

### 2.1. Criteria for including Studies

#### 2.1.1. Types of Studies

We will include published randomised controlled trials (RCTs) focused on BJOEI for treating GC. Language restrictions include English and Chinese.

#### 2.1.2. Types of Participants

This study will include RCTs in which participants were confirmed to have GC by biopsy and postoperative pathological examination. There were no limitations in age, sex, race, course, and severity of disease.

#### 2.1.3. Types of Interventions

This study will include interventions in patients who received the typical dosage of BJOEI, 10–30 ml (diluted with 250 ml sterilised normal saline) once per day through an intravenous drip. The BJOEI group referred to BJOEI combined with the same interventions as the control group. Studies that administered BJOEI to the control group as an adjunctive therapy will not be included.

#### 2.1.4. Types of Control Groups

We will include studies in which the control group adopted standard treatment (e.g., chemotherapy and radiotherapy).

#### 2.1.5. Outcome Measures

1. Primary outcomes: the primary outcomes of this study were clinical total effective rate, performance status, and ADRs
2. Secondary outcomes: safety of the BJOEI therapies, including adverse events and withdrawals for any reason

### 2.2. Search Methods for Identification of Studies

#### 2.2.1. Data Sources

We will search relevant databases from inception to 31 July 2021: PubMed, EMBASE, CENTRAL, Web of Science, Chinese Biomedical Literature Database (CBM), China National Knowledge Infrastructure (CNKI), Wanfang Database, and Chinese Scientific Journals Database (VIP Database).

#### 2.2.2. Other Search Resources

We will search clinical trial databases such as the Chinese Clinical Trial Registry (ChiCTR) and https://ClinicalTrials.gov for more data.

#### 2.2.3. Search Strategy

The combination of MeSH terms and text words was applied for retrieval. “Stomach Neoplasms” was regarded as the MeSH term. All strategies were adapted from different databases. An example of a search strategy for the PubMed database shown in Table 1 will be modified and used for the other databases. The searching strategy in PubMed is presented in supplementary file S2.

### 2.3. Data Collection and Analysis

#### 2.3.1. Selection of Studies

**Figure 1:**
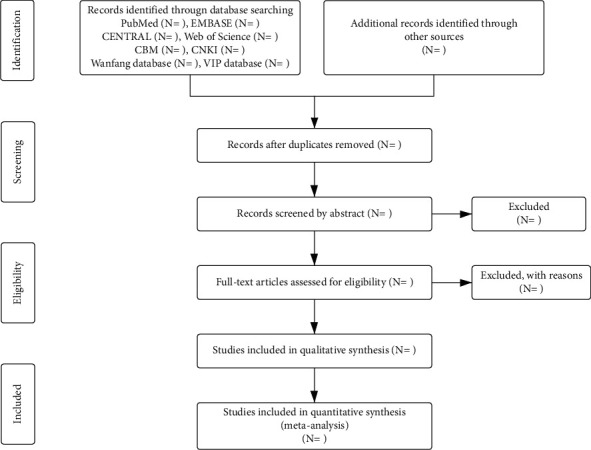
EMBASE = excerpt Medica Database, CENTRAL = PubMed Central, CBM = Chinese Biomedical Literature Database, and CNKI = China National Knowledge Infrastructure.

All data were extracted independently by two investigators (LC and XW), and any discrepancies between the reviewers were resolved by an intercessor (HW or JL) until a consensus was reached. A flowchart of this study is outlined in Figure 1.

#### 2.3.2. Data Extraction and Management

Data retrieved from the publications included author's name, year of publication, number of patients, average age, sex, dosage, course of treatment, and outcome data. When necessary and feasible, the corresponding authors of the selected studies will be contacted to obtain missing or incomplete data.

### 2.4. Synthesis of Data

A quantitative synthesis will be conducted for outcomes reported in more than one homogeneous RCT. The systematic review will be performed using RevMan 5 software. We will choose random- or fixed-effect models based on the analysis of heterogeneity. Randomised individuals will be considered in the unit of analysis issues. If a meta-analysis is not appropriate because of clinical/methodological issues or statistical heterogeneity, a narrative summary of the findings or relevant subgroup analyses will be used.

#### 2.4.1. Measures of Treatment Effect

For outcomes, this meta-analysis chose relative risk (RR) to evaluate dichotomous outcomes, while using mean difference (MD) to assess continuous variables. Each outcome numerical value was presented with 95% confidence intervals (95% CIs).

#### 2.4.2. Assessment of Publication Bias

If this systematic review will include 10 or more articles, funnel plots will be used to test the risk of publication bias.

#### 2.4.3. Heterogeneity Analysis

Heterogeneity between RCTs was analysed using the chi-square test and estimated using *I*^2^. Results of *P* ≥ 0.1 and *I*^2^ ≤ 50% suggested a lack of significant heterogeneity, and a fixed-effect model was used accordingly; otherwise, the random effects model was used.

#### 2.4.4. Subgroup Analysis

When conducting a meta-analysis, several subgroup analyses will be performed to identify subpopulations that may be associated with differences in Chinese medicine efficacy.

#### 2.4.5. Sensitivity Analysis

Trials with low risk of bias, unclear risk of bias, and high risk of bias will be synthesised separately. The results of the sensitivity analysis will be reported.

### 2.5. Quality Assessment

Two reviewers will present findings concerning the quality of evidence by independently assessing the quality of outcomes using the Grading of Recommendations Assessment approach. This assessment of evidence quality includes the risk of bias, heterogeneity, indirectness, imprecision, and publication bias. The quality of the evidence will be classified as high, moderate, low, or very low.

## 3. Discussion

GC, a common digestive tract tumour, is characterised by a high incidence, metastasis, and mortality rate, as well as a low early diagnosis, radical resection, and 5-year survival rate [41]. Modern cancer treatments aim to prolong life, whereas patients often wish to pursue quality of life. Historically, TCM has been in practice for thousands of years and has an irreplaceable role in complementary and adjuvant therapy. An increasing number of studies have shown that TCM formulas or monomers combined with modern treatment can be beneficial for patients by reducing adverse reactions, enhancing immune function, delaying disease progression, and inhibiting recurrence and metastasis [42–44]. According to TCM theory, *Brucea javanica* has heat-clearing (*Qingre* in Chinese *Pinyin*), detoxifying (*Jiedu* in Chinese *Pinyin*), and malaria-preventing (*Jienue* in Chinese *Pinyin*) properties. In recent years, BJOEI, a new generation of antitumour drugs extracted from *Brucea javanica,* has been widely used in the treatment of GC [45, 46]. However, the quality of numerous randomised controlled trials is inconsistent, and the efficacy and safety of BJOEI remain controversial. Therefore, it is necessary to systematically evaluate the efficacy and safety of BJOEI for the treatment of GC. Although some scholars have previously conducted a systematic review of BJOEI in the treatment of GC, no clear conclusions have been drawn. The quality of the synthesised evidence has not yet been evaluated. In this study, we analysed different standard treatments (such as different chemotherapy) to evaluate the clinical efficacy of BJOEI, with the aim of providing powerful evidence for clinicians to apply adjuvant therapy for patients with gastric cancer.

## Figures and Tables

**Table 1:** Searching strategy in PubMed.

Search strategy

#1 “Stomach Neoplasms” [Mesh]
#2 “Stomach Neoplasms *∗*” [Title/abstract] OR “Gastric Cancer *∗*” [Title/abstract] OR “Gastric Carcinoma” [Title/abstract] OR “Gastric Neoplasm *∗*” [Title/abstract] OR “Cancer of Stomach” [Title/abstract] OR “Stomach Cancer *∗*” [Title/abstract]
#3 #1 OR #2
#4 “Javanica oil emulsion injection” [Title/abstract] OR “Yadanzi” [Title/abstract] OR “*Brucea javanica* oil emulsion” [Title/abstract] OR “*Brucea javanica*” [Title/abstract]
#5 #3 AND #4

## Data Availability

The data and materials are available from the corresponding authors upon request.

## Supplementary Materials

Supplementary Materials S1: PRISMA 2009 checklist. S2: searching strategy in PubMed.
