# Supplementary material for: Efficacy and Safety of Brucea javanica Oil Emulsion Injection for Treating Gastric Cancer: A Protocol for a Systematic Review and Meta Analysis
Source: Evid Based Complement Alternat Med. 2021 Oct 15;2021:5236454. doi: 10.1155/2021/5236454 (PMC8536405; doi:10.1155/2021/5236454)
Supplement: Supplementary Materials — S1: PRISMA 2009 checklist. S2: searching strategy in PubMed. [file 5236454.f1.zip › 5236454.f1/supplementary file S2-Searching strategy in PubMed.doc]

**Searching strategy in Pubmed**

**Pubmed:** ("Stomach Neoplasms" [Mesh] OR "Stomach Neoplasms∗" [Title/Abstract] OR "Gastric Cancer∗" [Title/Abstract] OR "Gastric Carcinoma" [Title/Abstract] OR "Gastric Neoplasm∗" [Title/Abstract] OR "Cancer of Stomach" [Title/Abstract] OR "Stomach Cancer∗" [Title/Abstract]) AND ("Javanica oil emulsion injection" [Title/Abstract] OR "Yadanzi" [Title/Abstract] OR "Brucea javanica oil emulsion" [Title/Abstract] OR "Brucea javanica" [Title/Abstract])
